# Supplementary material for: Functional Characterization of Accessible Chromatin in Common Wheat
Source: Int J Mol Sci. 2024 Aug 29;25(17):9384. doi: 10.3390/ijms25179384 (PMC11395023; doi:10.3390/ijms25179384)
Supplement: Supplementary file 1 [file ijms-25-09384-s001.zip › ijms-3174057-supplementary.pdf]

Functional characterization of accessible chromatin in common wheat

Dongyang Zheng [1,†], Kande Lin [1,†], Xueming Yang [2], Wenli Zhang [1,*] and Xuejiao Cheng [1,*]

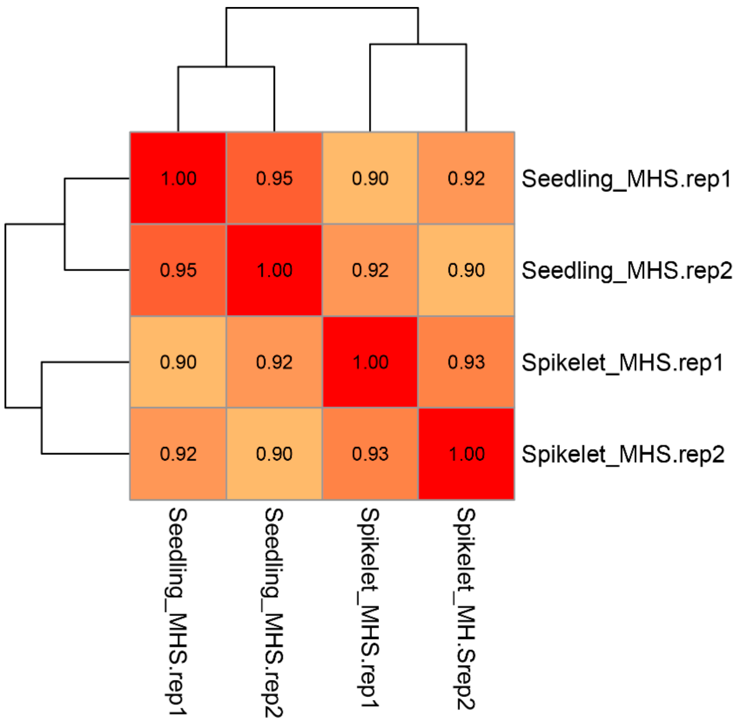

Figure S1. Correlation analyses of biologically replicated (MNase hypersensitive sequencing) MH-seq data sets generated from seedling and spikelet, respectively.

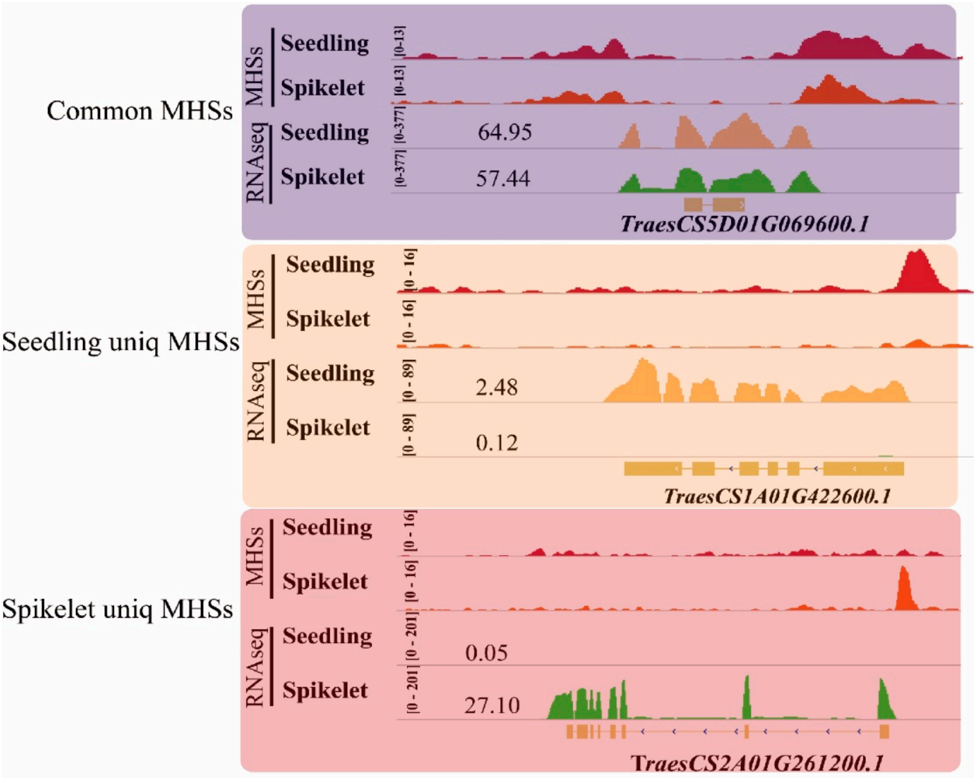

Figure S2. IGV snapshot illustrating subtypes of MNase hypersensitive sites (MHSs) between seedling and spikelet, i.e. common MHSs, seedling and spikelet unique MHSs.

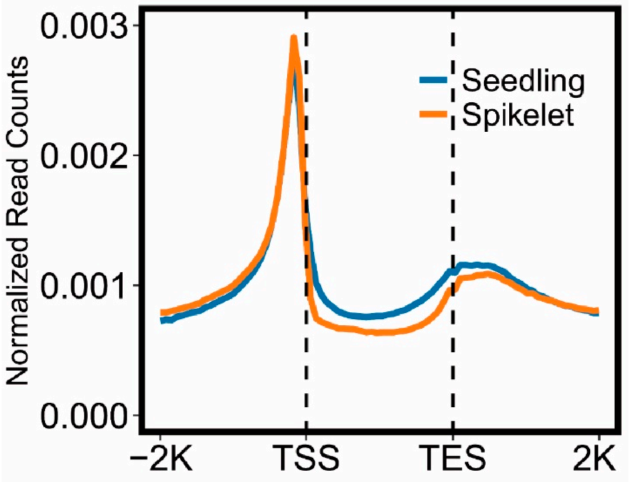

Figure S3. Comparison of normalized MHS read density between seedling and spikelet. Normalized MH-seq read counts were plotted across ± 2 kb from the transcription start sites (TSSs) to the transcription end sites (TESs) of genes in each tissue.

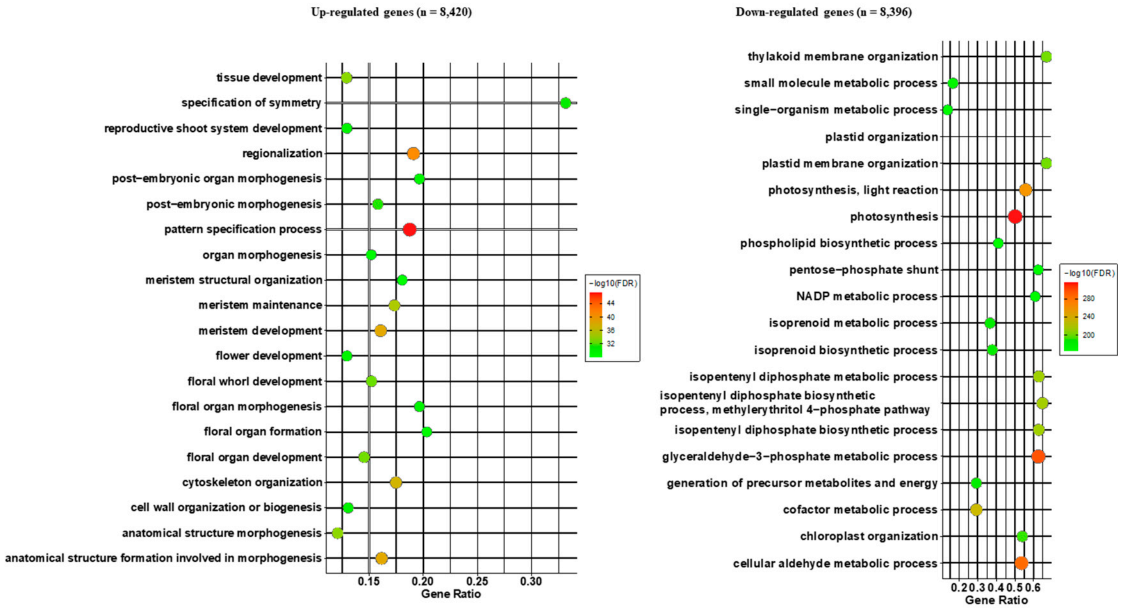

Figure S4. Gene Ontology (GO) enrichment analyses of differentially expressed genes (DEGs).

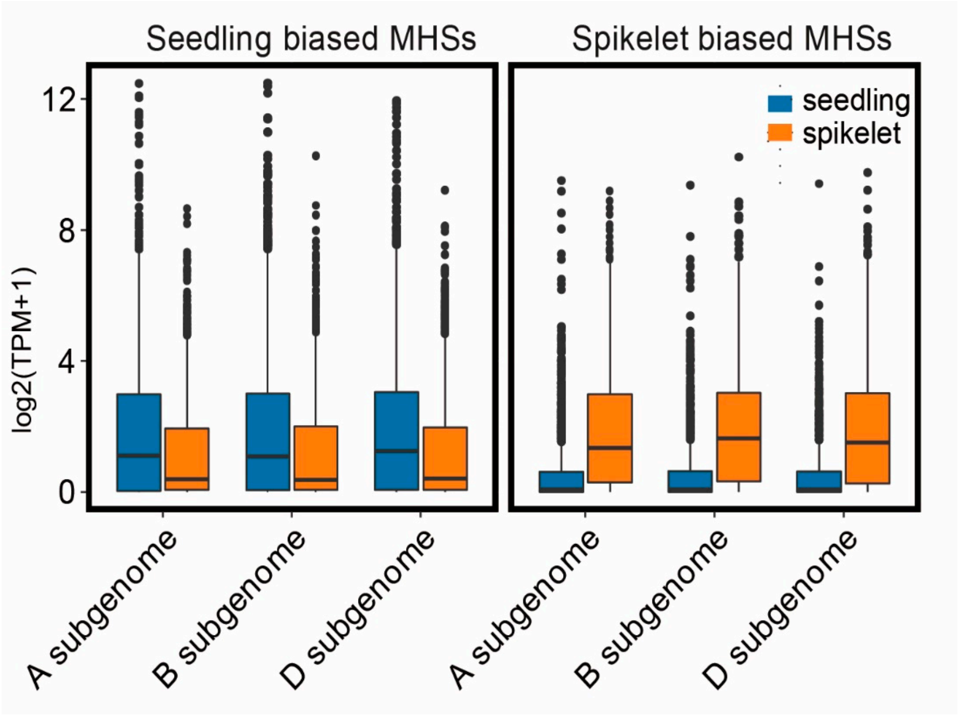

Figure S5. Comparison of expression levels of genes associated with tissue biased MHSs in each subgenome.

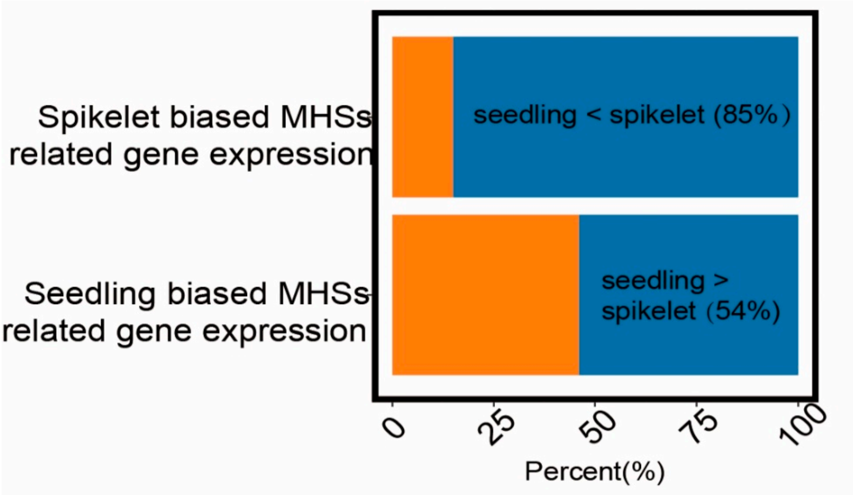

Figure S6. Percentage of expressed genes associated with tissue biased MHSs.

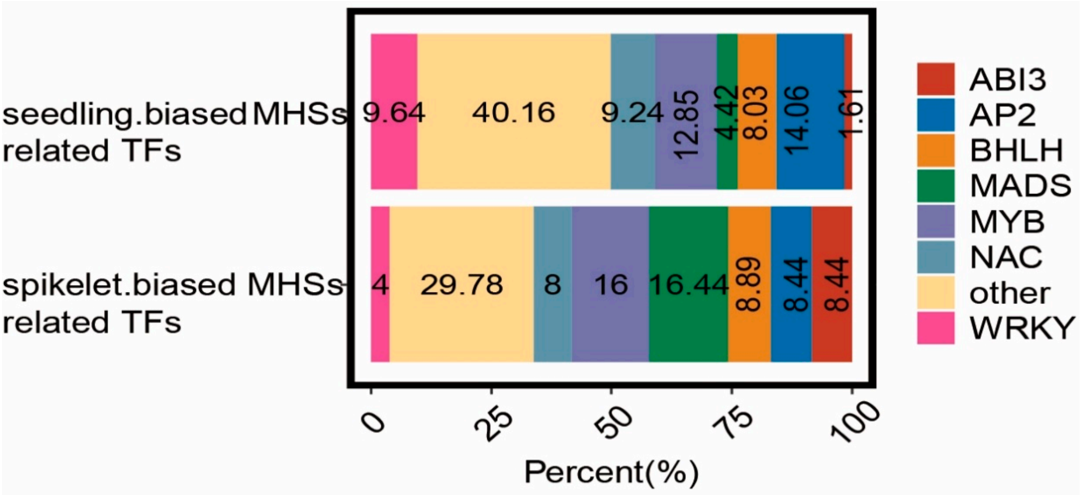

Figure S7. Percentage of transcription factors (TFs) (*MY*B, *MADS*, *WRKY, ABI3, AP2, BHLH, NAC* and others) associated with tissue biased MHSs.

Seedling	Spikelet	
0	0	TraesCS2A01G116900
0	0	TraesCS2B01G136100
0	0.099	TraesCS2D01G118200

Figure S8. Expression levels (TPM) of TF *WZFP* in A, B and D subgenome.

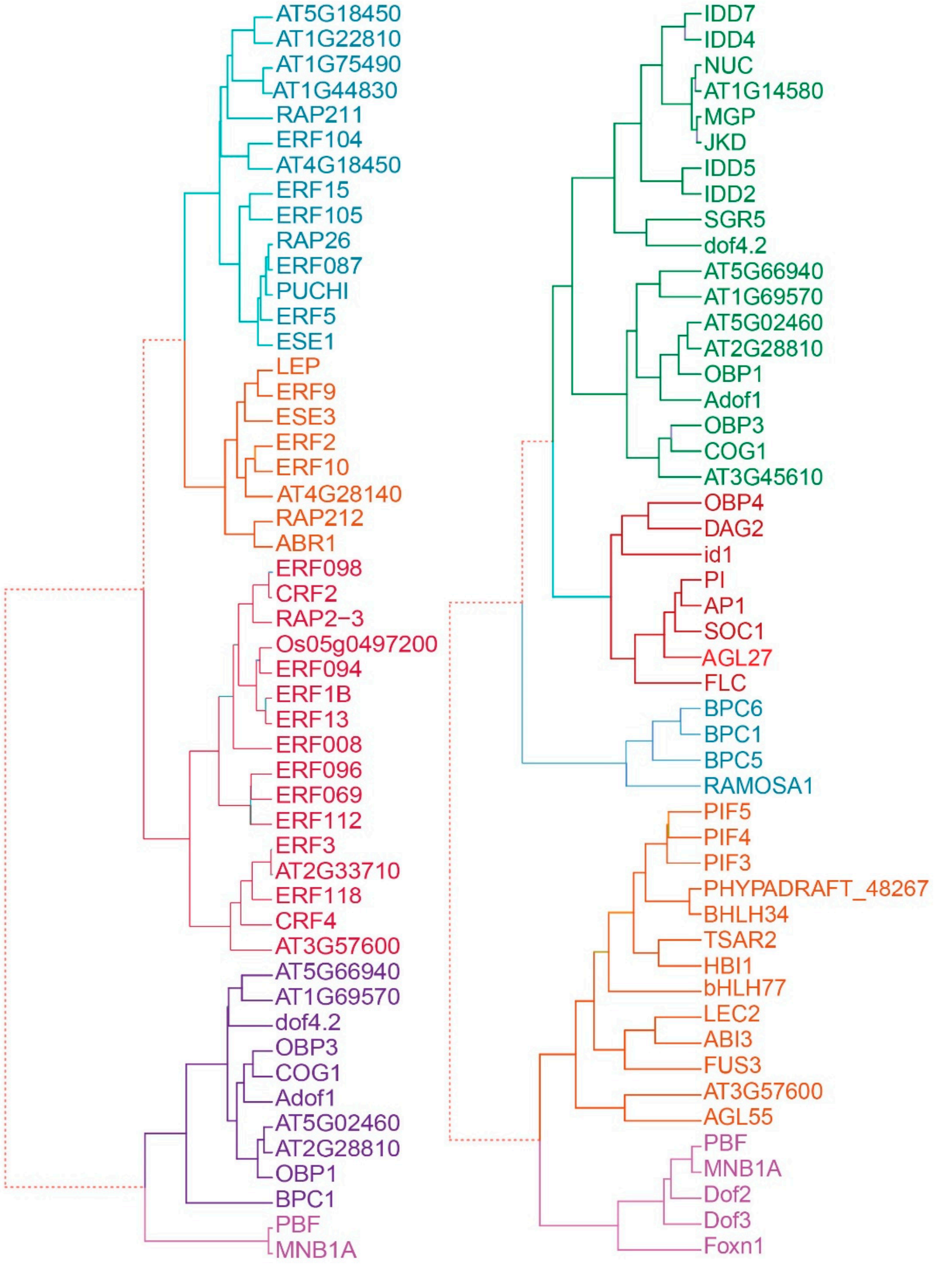

Figure S9. Dendrogram of seedling (left) and spikelet (right) biased TFs.

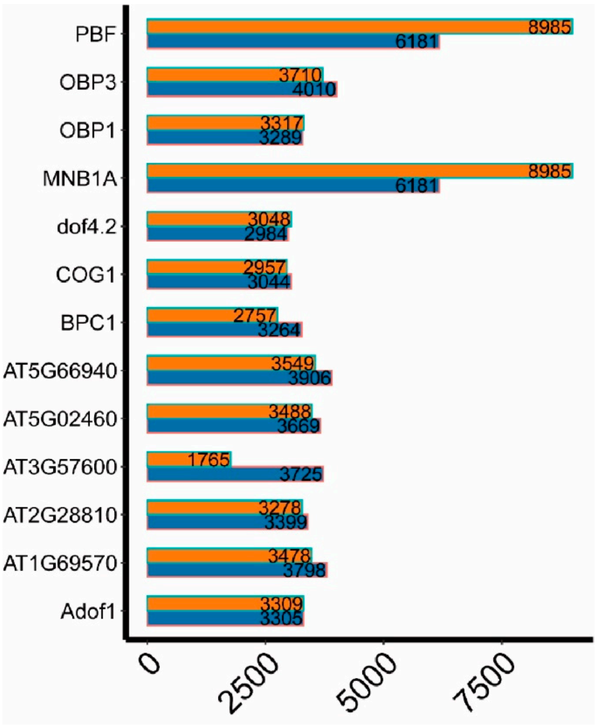

Figure S10. Number of transcription factor's binding sites (TFBS) shared between seedling and spikelet.

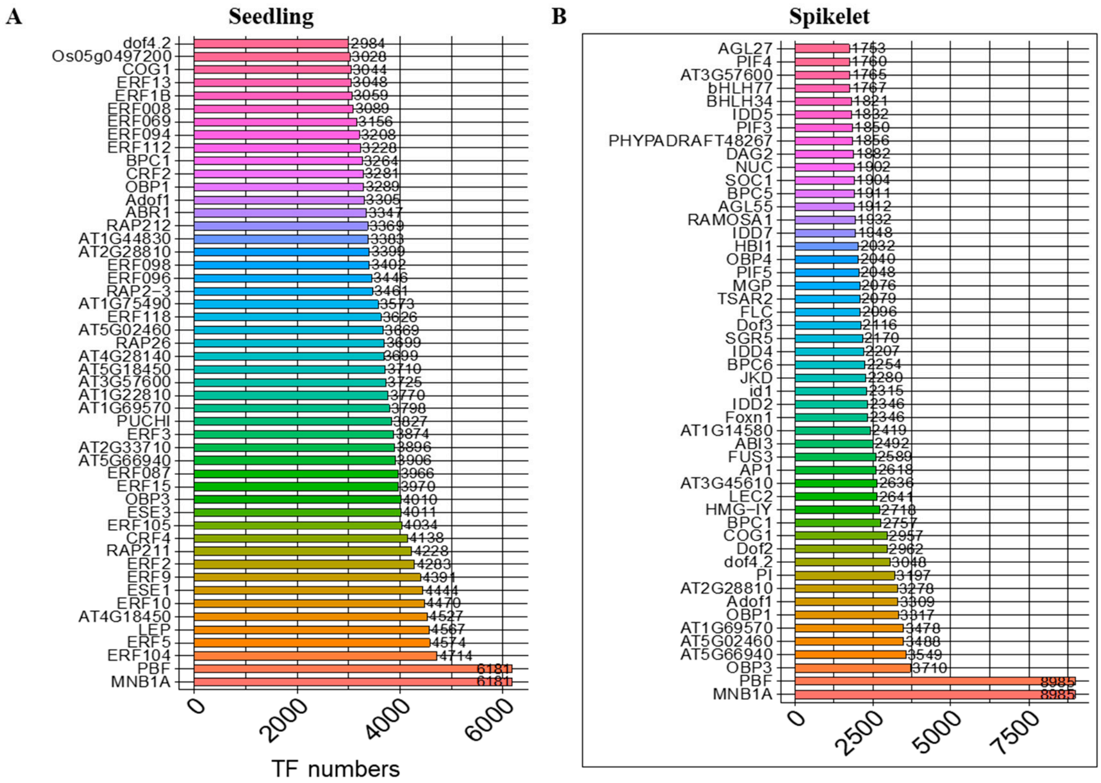

Figure S11. Number of the top 50 of divergent TFBS in seedling (A) and spikelet (B).

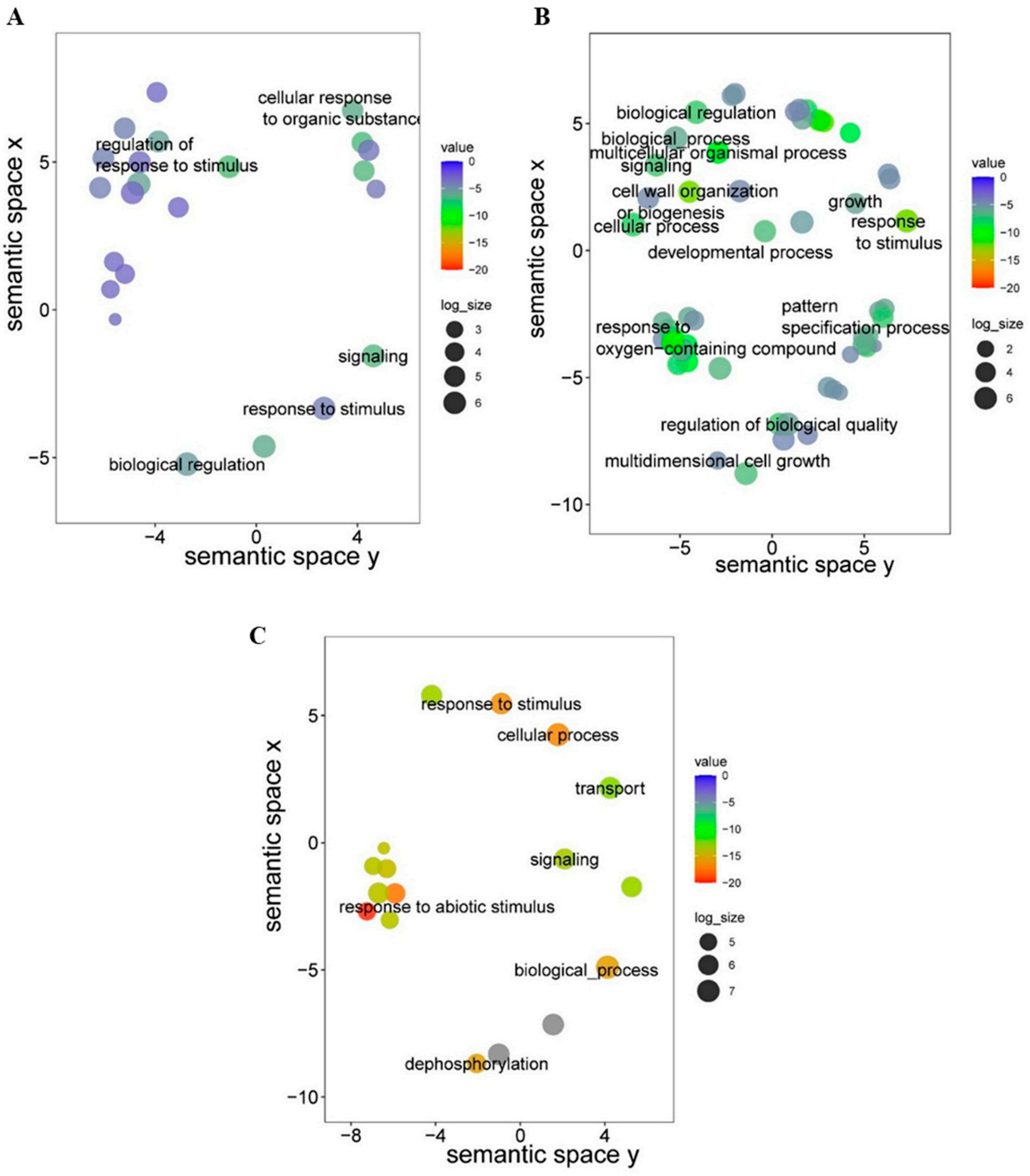

Figure S12. GO enrichment analyses of genes related to different types of footprints. (A) The overlapped TFs related genes between seedling and spikelet. (**B**) The top 50 TFs with the highest number of differences in the spikelet. (**C**) The genes corresponding to the top 50 TFs with the highest number of differences in the seedling

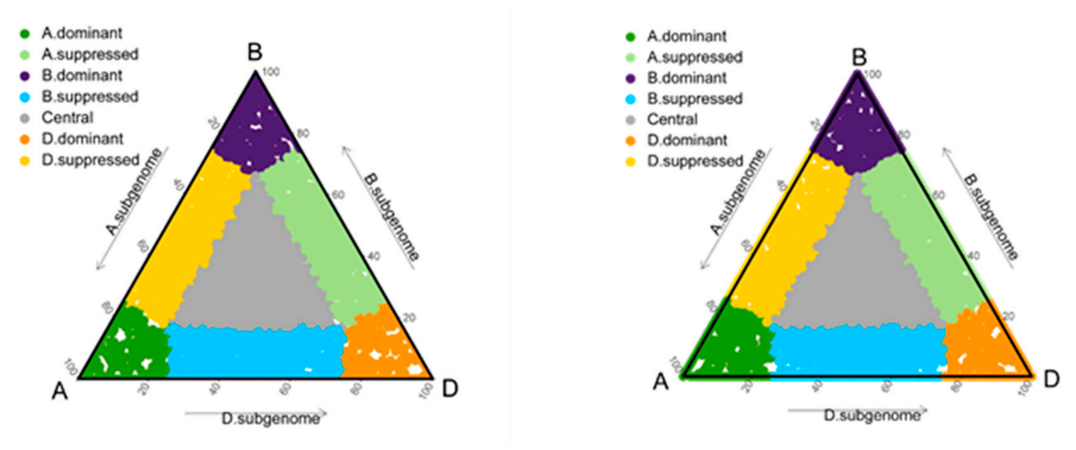

Figure S13. Number of TFBS in triad genes calculated by TOBIAS in seedling (A) and spikelet (B).

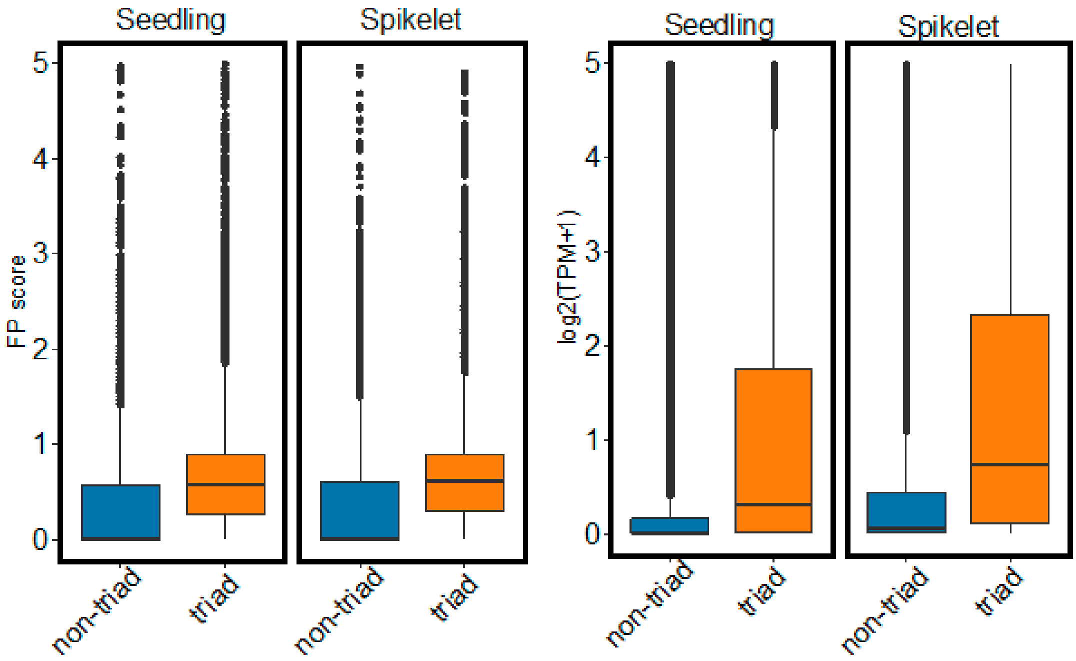

Figure S14. Comparison of the footprint (FP) score (A) and expression of triad and none triad genes (B) in two tissues.

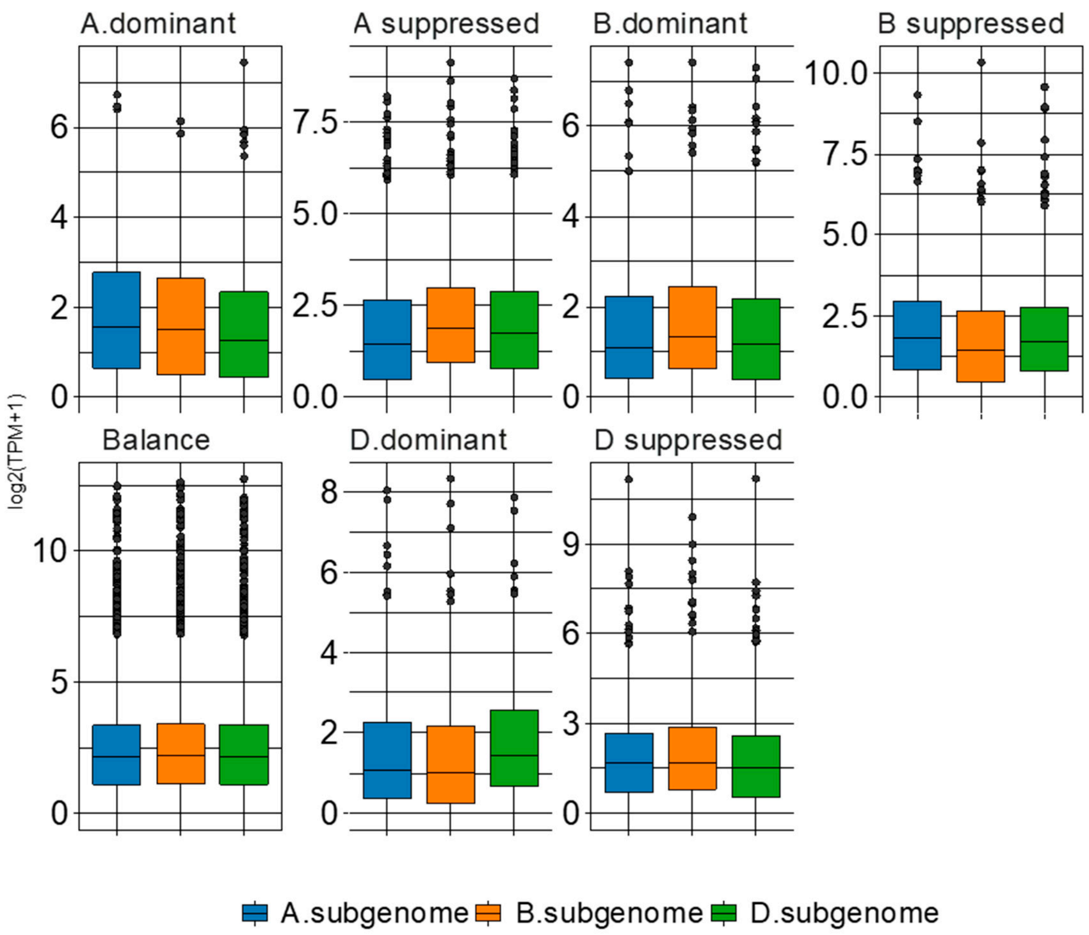

Figure S15. Expression levels of genes related to subgenome biased footprints in seedling.

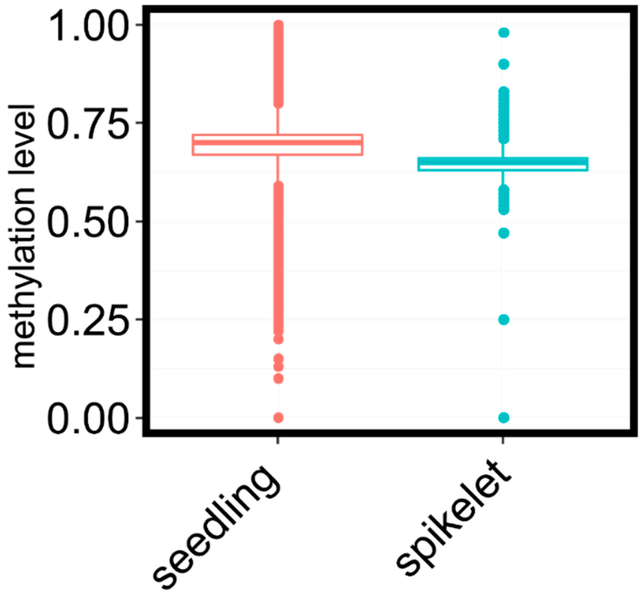

Figure S17. Comparison of DNA methylation levels between seedling and spikelet.

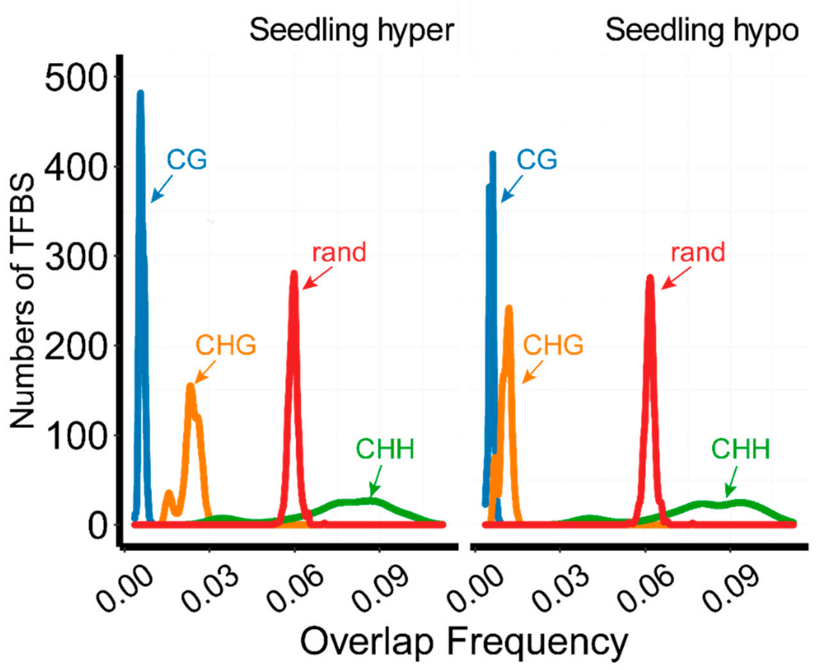

Figure S18. Statistics of overlapping frequency of TFBS with each type of DNA methylation region within ± 50 bp.

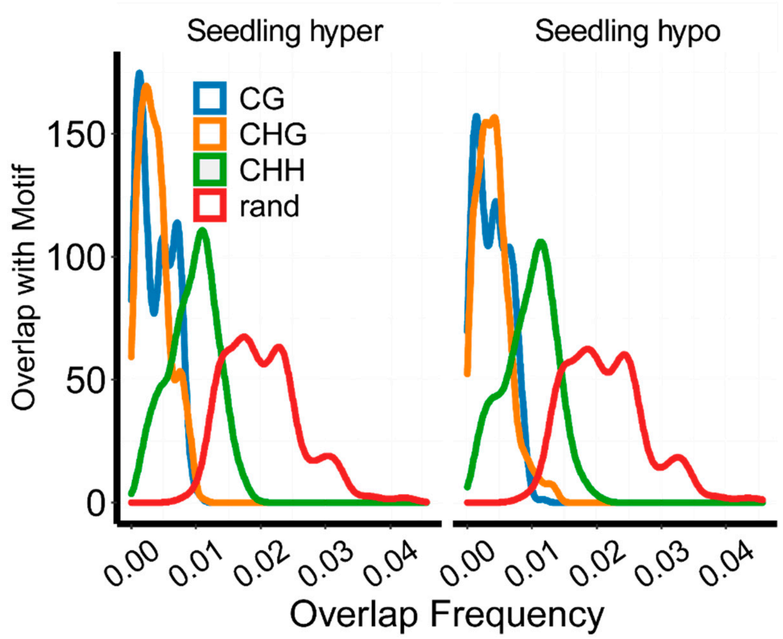

Figure S19. Statistics of overlapping frequency between DMSs in each cytosine context and TFBS.

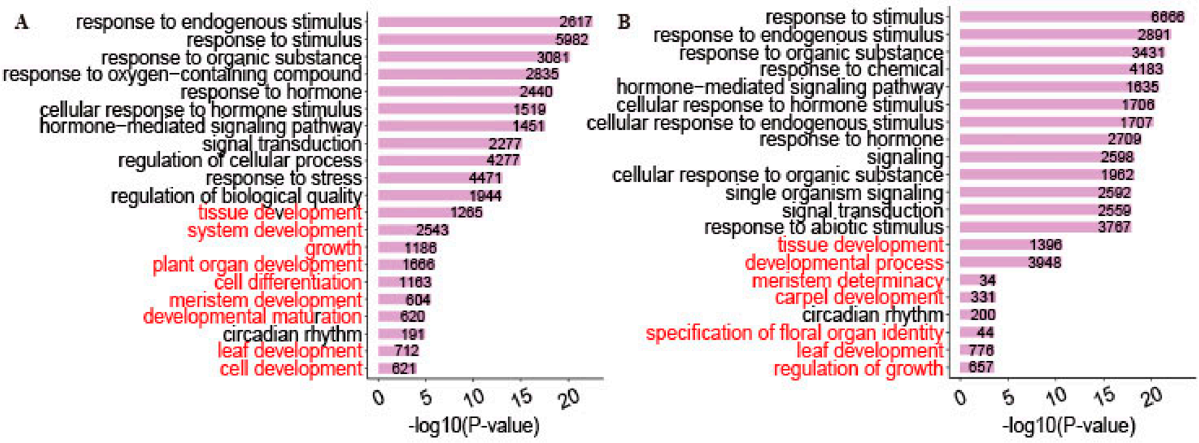

Figure S20. **GO enrichment analyses of genes that are the closest to the differential footprint and differential DNA methylation regions (DMRs) overlapping regions.** (A) seedling hyper DMR related genes. (B) seedling hypo DMR related genes.

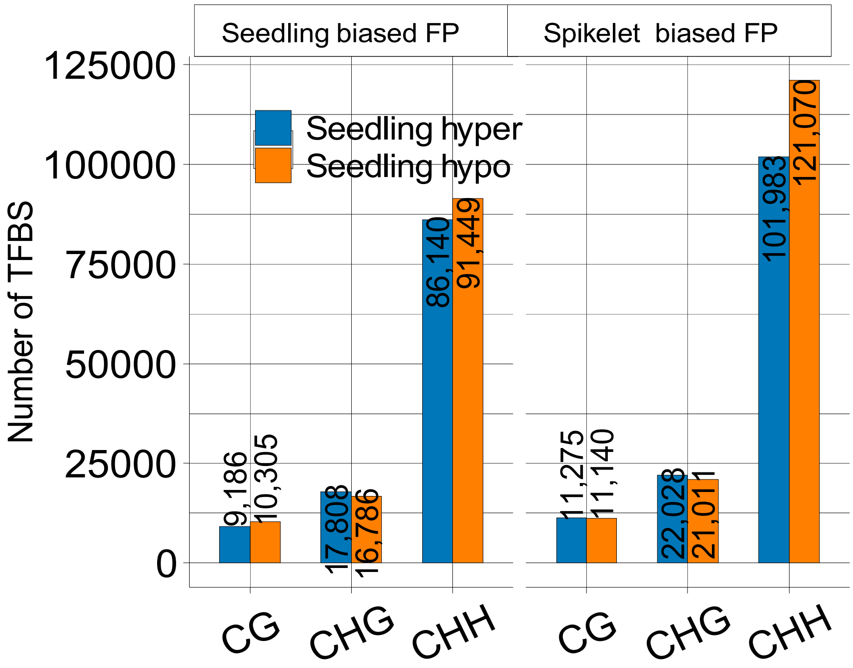

Figure S21. Number of overlaps between different types of DNA methylation and biased footprints.

Table S1. Summary of subtypes of MNase hypersensitive sites (MHSs) distributed in each chromosome

Chromosomes	Total MHSs in Seedling	Total MHSs in Spikelet	Seedling uniq MHSs	Spikelet uniq MHSs	Common MHSs
**chr1A**	11,980	11,527	4,430	3,815	7,550
**chr1B**	13,634	12,833	5,197	4,207	8,437
**chr1D**	11,479	11,371	4,022	3,745	7,457
**chr2A**	15,664	14,912	5,688	4,658	9,976
**chr2B**	16,817	16,218	6,109	5,257	10,708
**chr2D**	15,048	14,893	5,243	4,822	9,805
**chr3A**	14,587	13,836	5,353	4,350	9,234
**chr3B**	16,253	15,233	6,312	5,072	9,941
**chr3D**	14,279	13,900	5,173	4,559	9,106
**chr4A**	13,557	12,616	5,342	4,196	8,215
**chr4B**	11,519	10,832	4,181	3,317	7,338
**chr4D**	9,952	9,735	3,429	3,064	6,523
**chr5A**	14,704	14,097	5,353	4,504	9,351
**chr5B**	14,957	14,331	5,529	4,717	9,428
**chr5D**	13,889	13,489	4,945	4,370	8,944
**chr6A**	11,363	10,846	4,229	3,499	7,134
**chr6B**	13,732	12,928	5,282	4,300	8,450
**chr6D**	10,482	10,129	3,833	3,331	6,649
**chr7A**	15,402	14,653	5,967	4,993	9,435
**chr7B**	14,922	13,917	5,733	4,543	9,189
**chr7D**	15,226	14,823	5,697	5,082	9,529
**Total**	**289,446**	**277,119**	**107,047**	**90,401**	**182,399**

Table S2. Statistics of differential DNA methylation regions (DMRs)

Methylated cytosine context	Seedling hyper	Seedling hypo
**CG**	22,306 (0.4)	239,57 (0.44)
**CHG**	128,363 (2.72)	278,558 (5.12)
**CHH**	4,560,629 (96.8)	5,139,758 (94.44)

Table S3. Statistics of differential DNA methylation sites (DMSs)

Methylated cytosine context	Seedling hyper	Seedling hypo
**CG**	337,273 (1.14)	311,068 (0.96)
**CHG**	1,755,719 (5.93)	2,941,564 (9.07)
**CHH**	27,490,937 (92.93)	29,196,897 (89.98)

Table S4. Summary of differentially expressed genes (DEGs) associated with biased footprint (FP) and DMRs

Differential DNA methylation	Seedling biased	Spikelet biased
**Seedling hyper**	155/566	465/227
**Seedling hypo**	152/579	531/236
